# Assessment of platelet function in patients with stroke using multiple electrode platelet aggregometry: a prospective observational study

**DOI:** 10.1186/s12883-016-0778-x

**Published:** 2016-12-09

**Authors:** Ahmed Sabra, Sophia N. Stanford, Sharon Storton, Matthew Lawrence, Lindsay D’Silva, Roger H. K. Morris, Vanessa Evans, Mushtaq Wani, John F. Potter, Phillip A. Evans

**Affiliations:** 1 Medical School, Swansea University, Swansea, UK; 2 NISCHR Haemostasis Biomedical Research Unit, Morriston Hospital, Swansea, UK; 3 Cardiology Department, Singleton Hospital, ABMU Health Board, Swansea, UK; 4 School of Applied Sciences, Cardiff Metropolitan University, Cardiff, UK; 5 Department of Stroke Medicine, Morriston Hospital, Swansea, UK; 6 Norwich Medical School, University of East Anglia, Norwich, UK; 7 Emergency Department, Morriston Hospital, ABMU Health Board, Swansea, SA6 6NL UK

**Keywords:** Ischaemic stroke, Multiple electrode platelet aggregometry, Platelet function, Antiplatelet therapy, Aspirin, Clopidogrel, Aspirin resistance

## Abstract

**Background:**

There is a link between high on-treatment platelet reactivity (HPR) and adverse vascular events in stroke. This study aimed to compare multiple electrode platelet aggregometry (MEA), in healthy subjects and ischaemic stroke patients, and between patients naive to antiplatelet drugs (AP) and those on regular low dose AP. We also aimed to determine prevalence of HPR at baseline and at 3–5 days after loading doses of aspirin.

**Methods:**

Patients with first ever ischaemic stroke were age and sex-matched to a healthy control group. Three venous blood samples were collected: on admission before any treatment given (baseline); at 24 h and 3–5 days after standard treatment. MEA was determined using a Mutliplate® analyser and agonists tested were arachidonic acid (ASPI), adenosine diphosphate (ADP) and collagen (COL).

**Results:**

Seventy patients (mean age 73 years [SD 13]; 42 men, 28 women) were age and sex-matched to 72 healthy subjects. Thirty-three patients were on antiplatelet drugs (AP) prior to stroke onset and 37 were AP-naive. MEA results for all agonists were significantly increased in AP-naive patients compared to healthy subjects: ADP 98 ± 31 vs 81 ± 24, *p* < 0.005; ASPI 117 ± 31 vs 98 ± 27, *p* < 0.005; COL 100 ± 25 vs 82 ± 20, *p* < 0.005. For patients on long term AP, 33% (10/30) of patients were considered aspirin-resistant. At 3–5 days following loading doses of aspirin, only 11.1% were aspirin resistant based on an ASPI cut-off value of 40 AU*min.

**Conclusions:**

Many patients receiving low dose aspirin met the criteria of aspirin resistance but this was much lower at 3–5 days following loading doses of aspirin. Future studies are needed to establish the causes of HPR and potential benefits of individualizing AP treatment based on platelet function testing.

## Background

Platelets play a major role in arterial thrombus formation and therefore in the pathophysiology of ischaemic stroke [1–4]. Excessive platelet activation leads to increased thrombin generation and potentially abnormal thrombus formation [5]. Hence the importance of oral antiplatelet drugs, which are the mainstream therapy in the primary and secondary prevention of cerebrovascular disease [6, 7]. Currently, the antiplatelet (AP) drugs most widely used are aspirin and clopidogrel, and multiple electrode platelet aggregometry (MEA) has been used to study their effects on platelet function [8–11]. MEA has also been used to investigate the effects of non-opioid analgesics [12], anticoagulants [13], antifibrinolytics [14] and temperature [15] on platelet aggregation.

Despite the effectiveness of aspirin in the primary and secondary prevention of atherothrombotic disease, patients continue to suffer recurrent thromboembolic vascular events whilst on AP treatment [16]. This recurrence has been associated with high on-treatment platelet reactivity (HPR), with up to more than 60% of subjects being reported to be resistant to antiplatelet therapy (aspirin or clopidogrel) [17, 18]. Although some studies showed a link between HPR and major adverse vascular events [19, 20], the use of platelet function analysis to detect and manage HPR continues to be debated. Most studies that investigated HPR were undertaken for patients already receiving antiplatelet therapy without assessing baseline platelet reactivity [19, 20], which may partly explain the conflicting results. We were therefore interested in whether patients have a higher platelet reactivity at baseline, which may contribute to the suboptimal response to treatment. Hence this study aimed to compare platelet function, as determined by MEA, in healthy subjects and stroke patients prior to treatment initiation and between patients naive to AP therapy and those on regular low dose aspirin or clopidogrel.

## Methods

### Study design

A prospective observational study to compare platelet function between age matched healthy controls and patients with ischaemic stroke using MEA; and between patients naive to and on baseline AP therapy.

### Patient population

First time ischaemic stroke patients were recruited upon their presentation to the Emergency Department of a large teaching hospital (ABMU Health Board, Swansea, UK). Once a provisional diagnosis of stroke was made by the care team, strict inclusion criteria were applied. The inclusion criteria including only adults (≥18 years), the clinical assessment of ischaemic stroke was based on clinical history, examination and neuroradiology, the diagnosis of stroke was further validated by a member of the research team using WHO diagnostic criteria [21]. Full informed conset was sought from the outset and for those unable to consent due to lack of mental capacity assent was sought from personal or professional legal representatives. Exclusion criteria included: previous stroke; reciveing anticoagulant therapy; use of nonsteroidal anti-inflammatory drugs (NSAIDs) aside from low-dose aspirin; suffering from a disease known to alter coagulation (e.g. liver disease, malignancy, renal failure) or imminent death. Investigators were blinded to the result of MEA testing. Stroke patients were compared to an age-matched control group recruited from a healthy local population who were subsequently tested at the Haemostasis Biomedical Research Unit (HBRU). The helathy volunteers were recruited via various advertising means including posters, internal email or direct invitations of staff and patients’ relatives. Healthy volunteers were recruited if over 18 years of age without any significant co-morbidities aside from mild conditions not likely to affect platelet function (e.g. mild asthma or dyspepsia) and they were excluded if they had taken any NSAIDs or other agents that could affect platelet activity in the 2 weeks prior to testing.

### Blood sampling and data collection

One venous blood sample was collected from stroke patients on admission before any treatment given (baseline). Blood samples were also collected at 24 h and 3–5 days after standard treatment (aspirin 300 mg daily, or thrombolysis followed by aspirin 300 mg after the 24-h imaging to rule out intracerebral bleeding). The first 2 mL blood was discarded. Blood was then collected in 3 mL tubes containing 25 μg/mL hirudin (Dynabyte, Munich, Germany) as per the manufacturer’s recommendations. Further blood samples were taken for Full Blood Count (FBC), Prothrombin Time (PT), activated partial thromboplastin time (APTT) and fibrinogen levels. FBC samples were analysed on a Sysmex XE 2100 (TOA Medical Electronics) automated haematology analyser and routine clotting testing was undertaken using Sysmex CA1500 analyser. Fibrinogen concentrations were measured by ‘clauss’ method. The analysers were calibrated according to manufacturer’s instructions and fibrinogen calibration was checked against the second International Fibrinogen Standard Version 4 (NIBSC code 96/612). All reagents were obtained from Dade Behring.

### Multiple electrode aggregometry

**Fig. 1:**
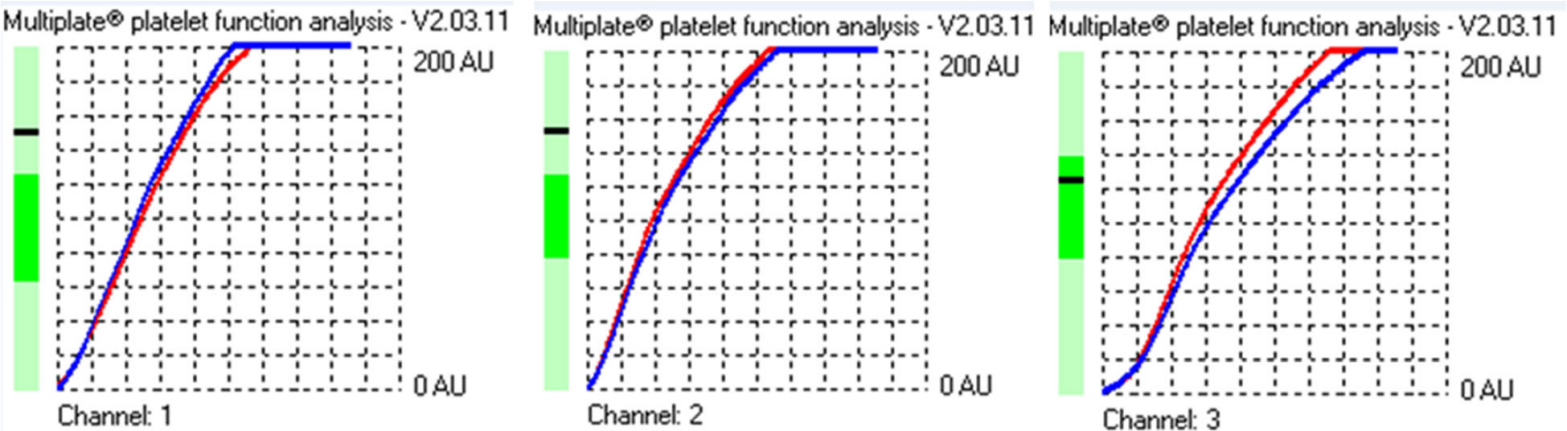
A graph of ADP (channel 1), ASPI (channel 2) and Collagen (channel 3) agonist testing in a patient with stroke. The aggregation is the increase of impedance during analysis. The velocity is the maximum slope of the aggregation curve. These 2 parameters determine the area under the curve from what the arbitrary aggregation units over time (AU*min) are calculated with values of between 0 and 200. The left side bar shows if values are within the manufacturer’s normal ranges. For this patient only collagen is within normal range but ADP and ASPI values are raised

Multiple electrode aggregometry (MEA) is a method that tests platelet function in whole blood, based on whole blood impedance aggregometry. In this study the Multiplate® analyser (Dynabyte GmBH, Munich, Germany) was used. The Multiplate® has five testing arease which can be loaded with the MEA test cells, each of the test cells has two independent sensor units which are made of two silver-coated, highly conductive copper wires. The Multiplate works by measuring platelet adhesion and aggregation to these conductive wires following activation of the platelets. As aggragtaion increases there is an increase in electrical impedance between the wires which is recorded on the Multiplate® device [22]. Platelet aggregation determined by MEA is calculated from the Area under the Curve (AUC) which is taken from the measured electrical impedance and quantified by arbitrary aggregation units over time (AU*min) (Fig. 1).

Hirudinated whole blood was kept at room temperature for 30 min before testing platelet aggregation using the Multiplate® analyser. A volume of 300 μl hirudinated blood was added to 300 μl of saline at 37 °C and allowed to incubate for 3 min in individual test cells. Then the following specific agonists were added to respective test cells and electrical impedance measured:

- Adenosine diphosphate (ADP, 20 μl of 0.2 mM stock solution) which triggers platelet activation via platelet ADP receptors (i.e. P2Y12 receptor that is inhibited by clopidogrel).
- ASPI test reagent (20 μl of 15 mM stock solution) contains arachidonic acid. This triggers platelet aggregation via platelet cyclooxygenase, which is blocked by aspirin.
- Collagen (20 μl of 100 μg/ml stock solution) which activates platelets via the collagen receptor.

HPR has been defined based on the manufacturer’s recommendations and published data with cut-offs of 50 AU*min for ADP-induced [19, 23] and 40 AU*min for ASPI-induced aggregation [24].

### Statistical analysis

To establish the baseline characteristics of the healthy and stroke groups descriptive analyses were performed. All the data was assessed for normality using normal probability plots and Shapiro–Wilk test of normality. Percentages are used to summerize categorical variables and chi-square tests were used to assess any difference between the groups. Continuous variables are presented as mean and standard deviation (SD) unless otherwise stated. Differences were assumed to be significant at 5% level. Two-sample Student’s *t*-test were used to compare differences between different groups. Any differences were then evaluated by using analysis of covariance (ANCOVA) whilst controlling for the effect of smoking. IBM SPSS Statistics for Windows, Version 22.0 (IBM Corp. Released 2013, Armonk, NY) was used to perform the analysis.

## Results

One hundred twenty two patients with suspected first-ever ischaemic stroke were recruited between May 2012 and February 2014. 52 patients were excluded. The reason for exclusion included: 18 had stroke mimics such as Bell’s palsy; sepsis and brain tumour; 8 transient ischemic attacks (TIA); 8 had a haemorrhagic stroke; 4 previous strokes; 3 cancers; 1 renal failure; 6 found to have been given loading aspirin prior to blood collection and 4 were taking NSAIDs such as Ibuprofen and Diclofenac. Leaving seventy patients (42 men and 28 women; mean age, 73.1 ± 13.3 years) with confirmed first time ischaemic stroke, these patients were used in the analysis. Their baseline characteristics are presented in Table 1. Stroke patients were matched for sex and age with 72 healthy subjects (mean age, 71.3 ± 6.9 years, *p* = 0.29; 40 men and 32 women, 0.59) recruited from the same population as the stroke patients. Whilst matched for age and sex the stroke group did have a significantly higher proportion of current smokers than healthy controls (22.9% versus 6.9%, *p* = 0.01).

**Table 1:** Baseline characteristics of patients with stroke (*n* =70)

Age, mean ± SD	73.1 ± 13.3
Sex: male/female	42/28
Current Smoker	16 (22.9%)
Hypertension	48 (68.6%)
Ischaemic heart disease	21 (30%)
Atrial fibrillation	18 (25.7%)
Diabetes mellitus	14 (20%)
Previous TIA	11 (15.7%)
Hyperlipidaemia	27 (38.6%)
Antiplatelets Aspirin only Clopidogrel only Both drugs	33 (47.1%) 26 3 4
Statins	26 (37.1%)
Glucose (mmol/L)	6.5 ± 1.7
Creatinine (μmol/L)	97 ± 27.2
D-dimer (ng/mL)	208 [104–488]
C-reactive protein (mg/L)	4 [1–10]
TOAST classification:	
Large artery Cardio-embolic Small vessel (lacunar) Undetermined aetiology	20 14 29 7

Values are presented as percentages, mean ± SD or median (interquartile range)

Full blood count and coagulation profile results are presented in Table 2 for all groups. Of the 70 patients, 31 were thrombolysed and 39 were loaded with 300 mg aspirin. At baseline 33 stroke patients were on antiplatelet therapy (26 on 75 mg of aspirin only, 3 on 75 mg of clopidogrel only and 4 on both agents) and 37 were AP-naive.

**Table 2:** Haematological tests for stroke patients and healthy subjects

Group	Healthy (*n* 72)	Stroke (*n* 70)		
		All	AP- naive	On AP
Plt (x10^9^/l)	245 ± 50	256 ± 79	271.3 ± 83	238 ± 71
Hb (g/dl)	14.3 ± 1.3	14.2 ± 1.7	14.4 ± 1.6	13.9 ± 2
HCT (g/l)	0.43 ± 0.04	0.42 ± 0.04	0.42 ± 0.04	0.41 ± 0.05
PT (secs)	10.6 ± 0.7	10.6 ± 0.6	10.5 ± 0.5	10.8 ± 0.7†
APTT (secs)	26 ± 2.3	24.2 ± 2.2*	23.9 ± 2*	24.4 ± 2.3*
FBG (g/l)	3.2 ± 0.5	3.8 ± 0.8*	3.9 ± 0.9*	3.7 ± 0.7*

Values reported as mean and standard deviation, *AP* antiplatelet naive at baseline

*p < 0.05 compared with healthy controls

†*p* < 0.05 compared with AP – naive group

Multiplate® analysis showed that ASPI was the only significant parameter that differed between the healthy subjects and the entire stroke group (97.6 ± 27.3 versus 81.1 ± 50.1, *p* = 0.02). These differences reversed when healthy subjects were only compared with AP-naive patients (97.6 ± 27.3 versus 117.4 ± 30.7, *p* < 0.005). In the AP-naive patients, significantly higher values were also observed for ADP and Collagen as shown in Table 3. All MEA parameters were significantly lower in patients who were on regular antiplatelet therapy at baseline as compared to healthy subjects and AP-naive patients (Table 3). All these differences continued to be significant after controlling for smoking status using ANCOVA.

**Table 3:** Results of MEA for stroke patients and healthy subjects

Group	Healthy (*n* 72)	Stroke (*n* 70)		
		All	AP-naïve (*n* 37)	On AP (*n* 33)
ADP	81.3 ± 24.3	89.8 ± 30.1	98.2 ± 31.1**	80.3 ± 26.4†
ASPI	97.6 ± 27.3	81.1 ± 50.1*	117.4 ± 30.7**	40.4 ± 33.2***‡
Collagen	82.4 ± 20.2	82.8 ± 29.1	99.5 ± 25**	64.1 ± 20.9***‡

Values reported as mean and standard deviation, *AP* antiplatelet naive at baseline

*p < 0.05 compared with healthy controls

**p < 0.005 compared with healthy controls

***p < 0.00005 compared with healthy controls

†*p* < 0.05 compared with AP – naive group

‡*p* < 0.0000005 compared with AP – naive group

### Prevalence of high on-treatment platelet reactivity (HPR)

Based on the cut off values for HPR, 10 patients (33%) on long term aspirin (baseline sample) were aspirin-resistant or low responders but only 4 had ASPI values > 60 AU*min. Of the seven patients on clopidogrel at baseline, 5 (71%) were clopidogrel-resistant (4 patients had ADP values above 85 AU*min).

**Fig. 2:**
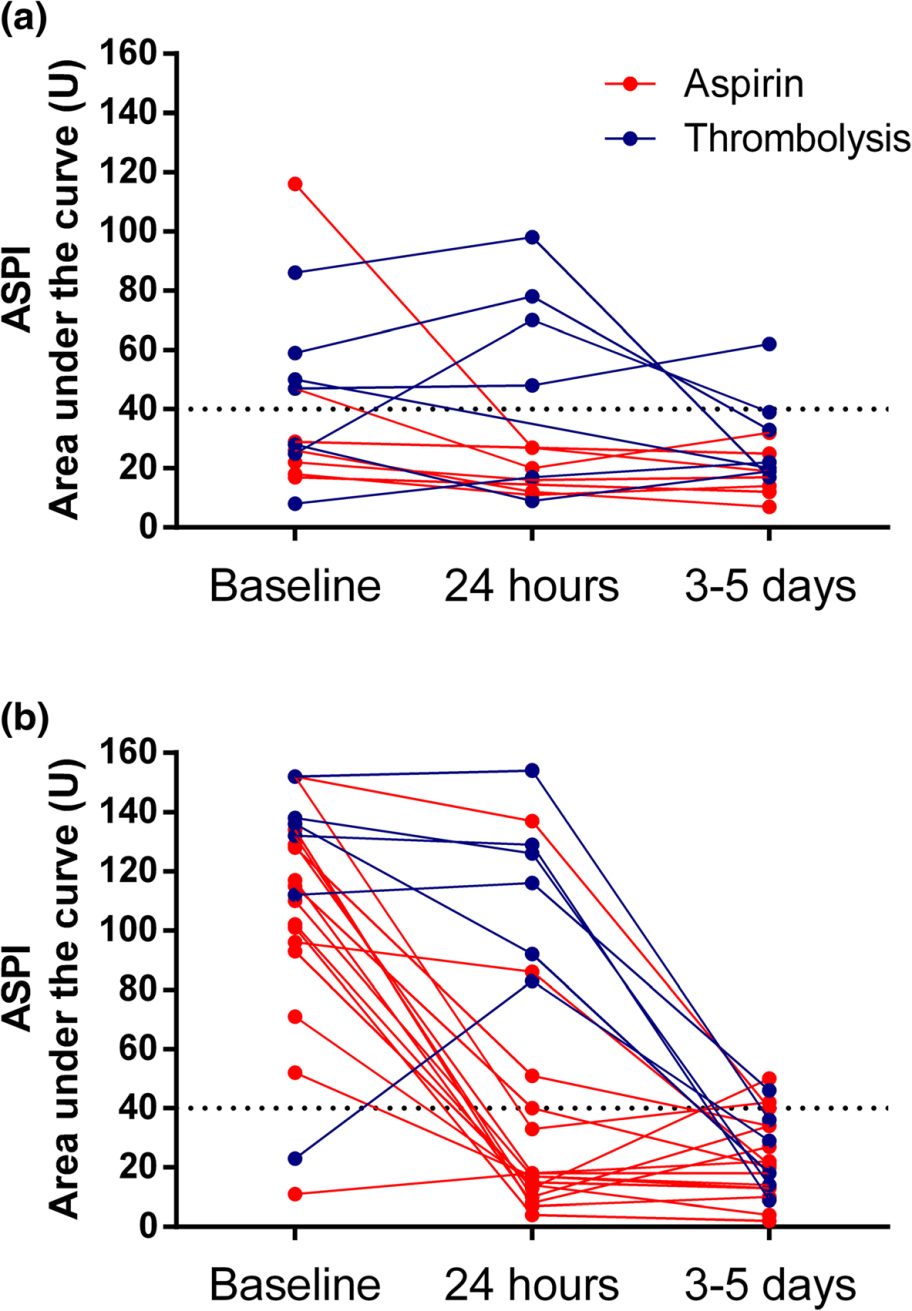
Distribution of ASPI-induced aggregation at baseline, 24 h and 3–5 days after 300 mg loading doses with aspirin. **a** In aspirin-naive patients, 13.6% had HPR (non-responder) at 3–5days. **b** Of patients on long term aspirin, only 7.1% were non responders at 3–5days. Horizontal dotted black lines indicate ASPI cut-off values for aspirin resistance, as reported in the literature

ASPI results were available for 36 patients at 3–5 days after 300 mg loading doses of aspirin. 13.6% (3/22) aspirin naive patients and 7.1% (1/14) on long term aspirin were considered aspirin-resistant but only one patient (1/36) had ASPI values > 60 AU*min. At 24 h, it is understandable that for thrombolysis patients (dotted lines, Fig. 2) ASPI values were higher than aspirin group (solid lines) because they have not received any aspirin pending the 24 h head CT scan to rule out intracerebral haemorrhage. The results of serial ASPI testing at baseline, 24 h and 3–5 days for patients who were aspirin-naive or on long term aspirin are shown in Fig. 2.

## Discussion

Our study is the first to assess platelet reactivity using MEA in patients with stroke at baseline before loading doses of 300 mg aspirin were given. The main finding showed that platelet aggregation in AP-naive patients is higher than in healthy subjects for all pathways investigated. However, it is difficult to ascertain if this is due to an underlying high platelet reactivity or is secondary to the acute event. Future larger studies are needed to confirm our findings and investigate this assumption.

Secondly, our study detected changes between long-term aspirin users and aspirin-naive patients. Despite the proved effectiveness of AP therapy, patients continue to have recurrent vascular events while on treatment [16]. In agreement with previously reported studies [25], about 50% of patients in our study were taking one or more AP drugs at presentation but this did not prevent stroke. New approaches to improve clinical outcomes used MEA to individualise antiplatelet therapy [19] and hence highlighting the importance of our findings. Based on definition of aspirin resistance in previous studies, 33.3% of patients would be defined as aspirin-resistant according to ASPI results. As more than 60% of long term aspirin users are considered responders, the use of loading dose of aspirin in these patients may not have any additional benefit. It may indicate the need to investigate thoroughly for underlying silent risk factors such as paroxysmal atrial fibrillation. The emergence of new P2Y12 antagonists and ongoing uncertainties of platelet function testing dictate the need for larger RCT to answer these questions and investigate the clinical benefit of AP switching strategy in stroke patients based on MEA results. This supports the recommendation by the National Institute for Health and Care Excellence for future research in this area about effectiveness and safety of clopidogrel, alone or in combination with aspirin, in treating stroke and TIA [26].

Thirdly, our study also detected the effect of aspirin loading therapy on platelet aggregation (ASPI) in acute stroke. Interestingly at 3–5 days after in hospital administration of aspirin, only 11.1% were non or low responders. Therefore, there is a discrepancy of more than 20% in presumed aspirin-resistance rates between self-reported and in hospital aspirin intake. Many factors may explain this discrepancy including poor compliance, timing of testing following aspirin intake and low-dose versus high-dose aspirin. Up to 50% of patients are reported to be poorly compliant [27, 28]. Although many strategies are in place to encourage the public to recognise and act on stroke symptoms, little has been done to improve compliance. Previous studies suggested female sex, single marital status, lower education level, depression, diabetes mellitus, polypharmacy, and smoking as predictors of poor compliance [27]. Hence, more educational initiatives targeting these high-risk patients of poor compliance should be done to enforce the importance of taking AP regularly. Another possible explanation of this discrepancy is the timing of baseline sample after aspirin intake, which was variable, whereas it was 2–4 h after administering aspirin for subsequent samples. Timing of platelet function testing has been acknowledged as a factor explaining the variability in the results with serum salicylate level peaking at 1 h after administration [5, 29]. The higher aspirin dose given acutely in hospital is another potential factor but published data do not support this assumption reporting that doses between 30 and 1300 mg per day have the same clinical efficacy [18, 30]. Nonetheless some may argue that this does not equate to similar platelet inhibition as detected by platelet function tests.

Despite the availability of a wide-range of techniques to investigate platelet function, their reliability has been questioned. A previous study showed poor correlation between six platelet function tests to define aspirin-resistance [31], and this variation in methodology may be a factor in explaining the difficulties in determining the true prevalence of AP resistance. Hence it is important to link these tests to clinical endpoints to establish cut-offs of each assay. Although LTA may be considered the gold standard for the determination of platelet activity, we chose to use Multiplate® in this study because it is a standardized fully automated point-of-care test that is relatively easy to use without many of the technical limitations of the previous assays [32]. Additionally the feasibility of using Multiplate® in the context of routine hospital service has been ascertained to provide reproducible and precise results in stroke patients [33]. This potentially makes our findings easier to replicate and translate into large clinical studies to investigate clinical endpoints.

Limitations: this is a small single centre study and hence may not be generalizable to other populations. Aspirin and clopidogrel intake at baseline was self-reported by patients or their family. Although MEA results indicate two thirds of the patients were taking aspirin, confirming compliance is an important issue but was beyond the scope of this study. Another limitation is the higher number of active smokers in the stroke group as compared to healthy subjects. Conflicting results exist about the effect of smoking on platelet function testing [20, 24, 34]. However in our study, ANCOVA analysis was used to control for the effect of smoking. Another limitation is the attrition rate despite the prospective study design. Multiplate analysis at 3–5 days was not possible for all cases for various reasons (patient discharged or transferred to another hospital, death, declined further samples, difficult to bleed). Despite the strict inclusion and exclusion criteria heterogeneity of stroke presentations and patient populations are key issues to consider when investigating stroke as noticed in this study, and subgroup analysis should be interpreted with caution.

## Conclusions

Despite these limitations our study reports a higher platelet reactivity as determined by Multiplate® in AP-naive stroke patients for all pathways as compared to healthy subjects. Many patients receiving low dose aspirin met the criteria of HPR or drug resistance but the prevalence was much lower at 3–5 days of inpatient treatment. It also reiterates the importance of compliance and probably timing of testing as potential factors affecting MEA testing. It also questions the benefits of giving aspirin in patients with laboratory evidence of response when this did not prevent recurrent stroke. Hence further work in large prospective multicentre trials is needed to confirm that the laboratory results can be translated onto clinical endpoints. For any future research the main question is whether the use of MEA to individualise AP treatment in stroke/TIA results in better clinical outcomes. We recommend establishing platelet reactivity at baseline to determine best AP therapy and then monitor their effect at 3 days to decide whether a switch is warranted. This may also help establish the true causes of HPR in stroke and the value of MEA in predicting future events.

## Abbreviations

ADP: Adenosine diphosphate
ANCOVA: Analysis of covariance
AP: Antiplatelet drugs
ASPI: Arachidonic acid
AU*min: Aggregation units over time
AUC: Area under the curve
COL: Collagen
HPR: High on-treatment platelet reactivity
LTA: Light transmission aggregometry
MEA: Multiple electrode platelet aggregometry
NSAIDs: Nonsteroidal anti-inflammatory drugs
RCT: Randomised control trial
SD: Standard deviation
TIA: Transient ischaemic attack
